# Supplementary material for: Intracellular magnesium optimizes transmission efficiency and plasticity of hippocampal synapses by reconfiguring their connectivity
Source: Nat Commun. 2024 Apr 22;15:3406. doi: 10.1038/s41467-024-47571-3 (PMC11035601; doi:10.1038/s41467-024-47571-3)
Supplement: Supplementary file 1 — Supplementary Information [file 41467_2024_47571_MOESM1_ESM.pdf]

Intracellular magnesium optimizes transmission efficiency and plasticity of hippocampal synapses by reconfiguring their connectivity

Hang Zhou[1, 2, ✉], Guo-Qiang Bi[1, 2, 3, 4], and Guosong Liu[5, 6 ✉]

[1] Faculty of Life and Health Sciences, Shenzhen University of Advanced Technology, Shenzhen, 518107, China
[2] Interdisciplinary Center for Brain Information, Brain Cognition and Brain Disease Institute, Shenzhen Institute of Advanced Technology, Chinese Academy of Sciences, Shenzhen, 518055, China
[3] Shenzhen-Hong Kong Institute of Brain Science, Shenzhen, 518055, China
[4] Hefei National Laboratory for Physical Sciences at the Microscale, and School of Life Sciences, University of Science and Technology of China, Hefei, 230031, China
[5] School of Medicine, Tsinghua University, Beijing, 100084, China
[6] NeuroCentria Inc., Walnut Creek, CA, 94596, United States

✉ To whom correspondence should be addressed:
zhouhang01@outlook.com, liu.guosong@gmail.com

Supplementary Information includes:

- **Supplementary Figures 1–11**
- **Supplementary Tables 1, 2**
- **Supplementary Notes**

Supplementary Figures, Tables and Notes

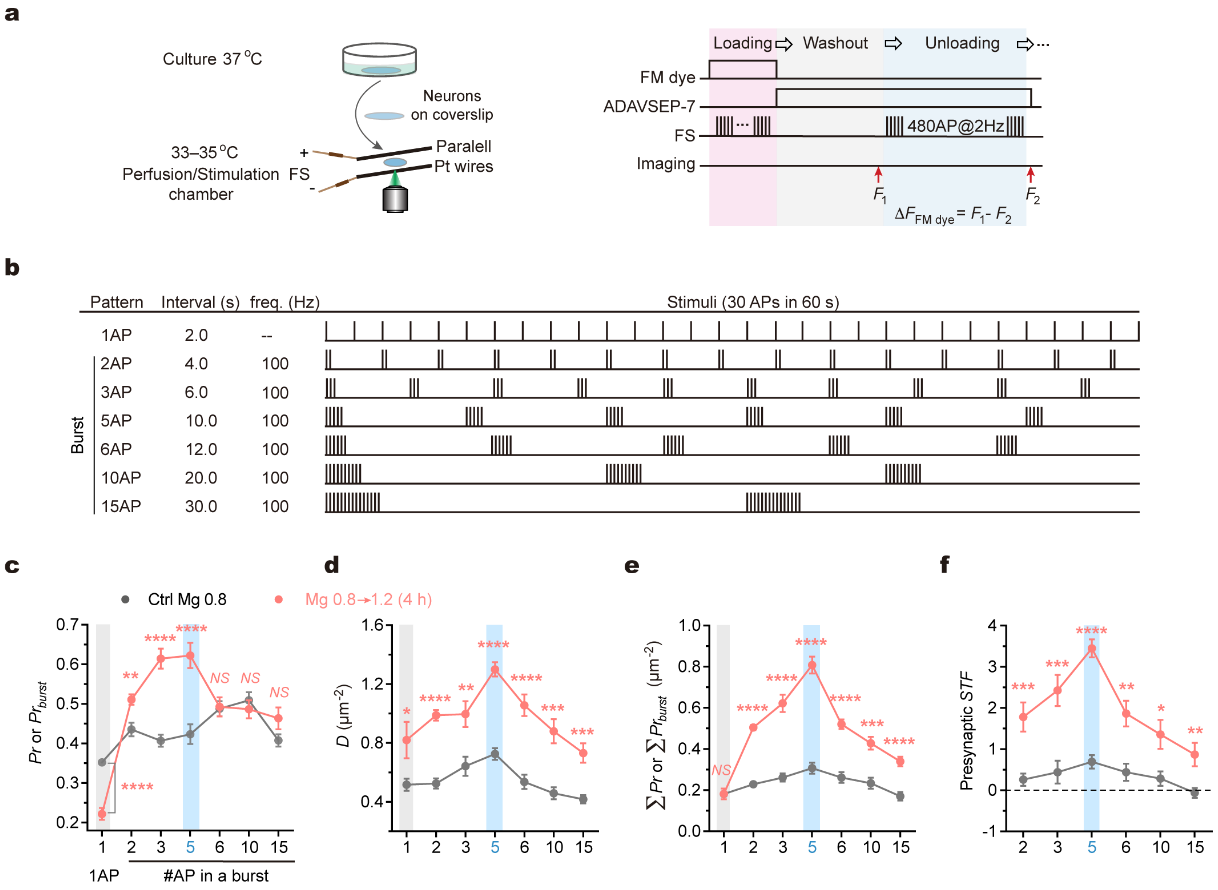

Supplementary Fig. 1 | Visualization of vesicle turnover in single boutons.

a, Experimental procedures for visualizing vesicle turnover in single boutons utilizing FM dye staining (for details, see **Methods**). **b,** Table of various patterns of field stimulation. To control total stimulating strength among various patterns, 30 APs were evenly assigned in 60 s. Frequency is set 100 Hz for all bursting patterns. **c–f,** Left to right**,** vesicular release probability, density of functional synapses, total presynaptic strength, and presynaptic short-term facilitation (STF, defined as $\mathbf{\Sigma} \boldsymbol{Pr_{burst}}/\mathbf{\Sigma} \boldsymbol{Pr}$) upon various patterns of inputs (*n* = 11 repeats for each group). *P* < 0.0001, = 0.0022, < 0.0001, < 0.0001, = 0.8798, 0.4658, 0.0924 in (**c**), *P* = 0.0310, < 0.0001, = 0.0041, < 0.0001, < 0.0001, = 0.0002, 0.0003 in (**d**), *P* = 0.9843, < 0.0001, < 0.0001, < 0.0001, < 0.0001, = 0.0001, < 0.0001 in (**e**), *P* = 0.0008, 0.0004, < 0.0001, = 0.0011, 0.0131, 0.0066 in (**f**). Data are shown as mean ± SEM. Two-sided student's *t* tests to compare physiological ($[Mg^{2+}]_o$ 0.8 mM) condition with elevated Mg^{2+} (0.8 to 1.2 mM for 4 h) condition. Significance: *NS*, no significance; * *P* < 0.05, ** *P* < 0.01, *** *P* < 0.001, **** *P* < 0.0001. Source data are provided as a Source Data file.

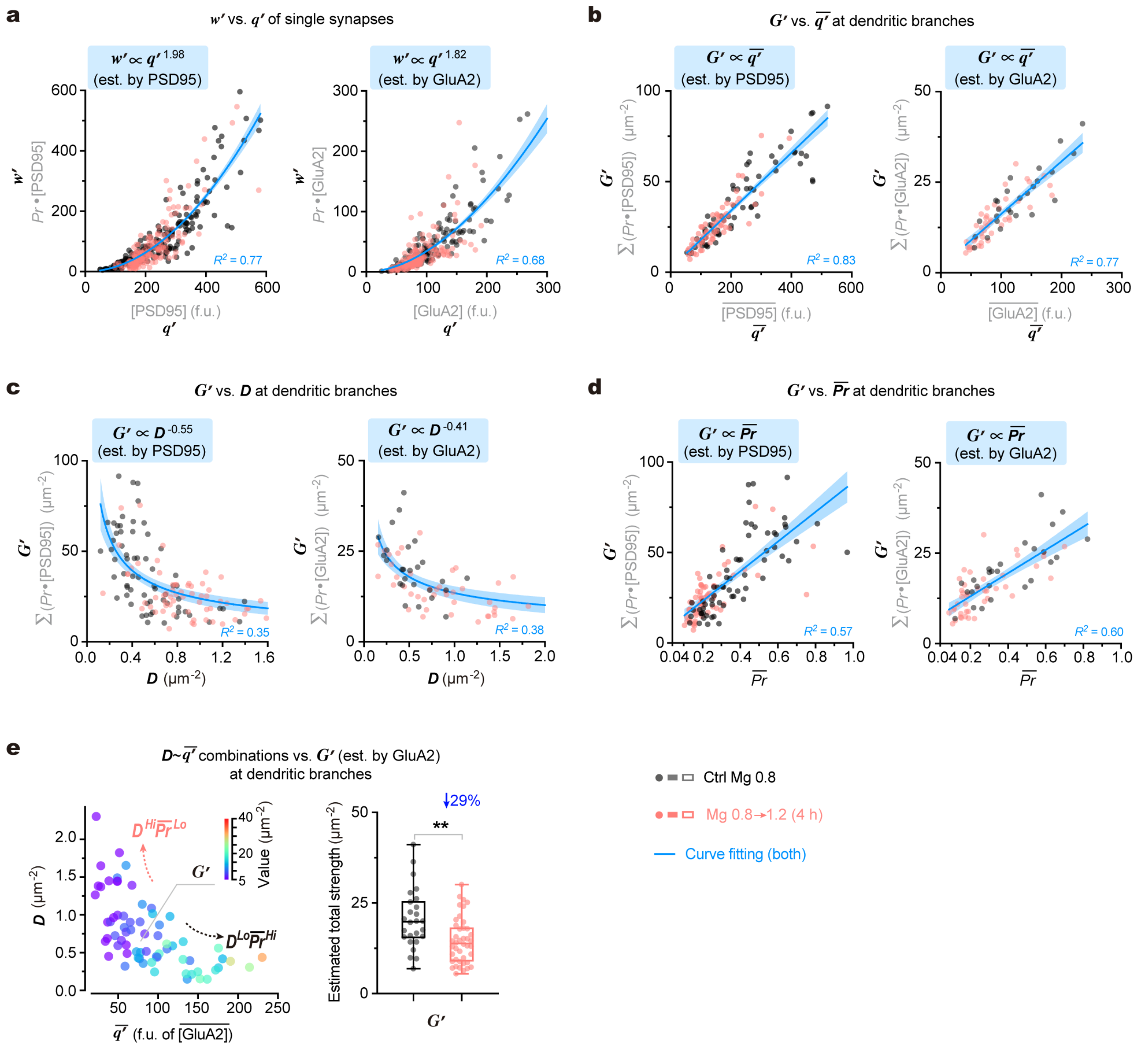

Supplementary Fig. 2 | Synaptic configuration determines transmission efficiency of synapses at dendrites. **a–d,** The same experiments as shown in **Fig. 2** examining transmission efficiency in basal transmission. **a,** Plots of estimated weight (***w′***) against estimated quantal size (***q′***) by [PSD95] (left) or [GluA2] (right) in single synapses (for PSD95, $n = 210$, 163 synapses from 4, 3 repeats; for GluA2, $n = 140$, 167 synapses from 3, 3 repeats). **b–d,** Plots of estimated total basal strength (***G′***) against the mean ***q′*** ($\overline{q'}$) or synapse density (***D***) or $\overline{Pr}$ at individual dendritic branches (for PSD95, $n = 65$, 62 branches from 4, 4 repeats; for GluA2, $n = 26$, 42 branches from 3, 3 repeats). The parameters were estimated by the product of ***Pr*** and the immunofluorescence of PSD95 (left) or GluA2*AMPAR (right). **e,** The relationship between ***D***, $\overline{q'}$ and ***G′*** (coded by pseudo color) at individual dendritic branches (***q′*** was approximated by [GluA2] at postsynaptic sites) and box-whisker plot of ***G′*** from physiological and elevated Mg^{2+} conditions ($n = 26$, 42 branches from 3, 3 repeats). Notably, this result was consistent with that using [PSD95] (**Fig. 2f, g**). Box borders and line, quantiles and median; whiskers, min and max. Grey axis labels, formulas for individual estimated parameters. In (**a–d**), blue lines/error bands, fitted curves/95% CIs. Two-sided Kolmogorov-Smirnov test (**e**), $^{**}P = 0.0086$. Source data are provided as a Source Data file.

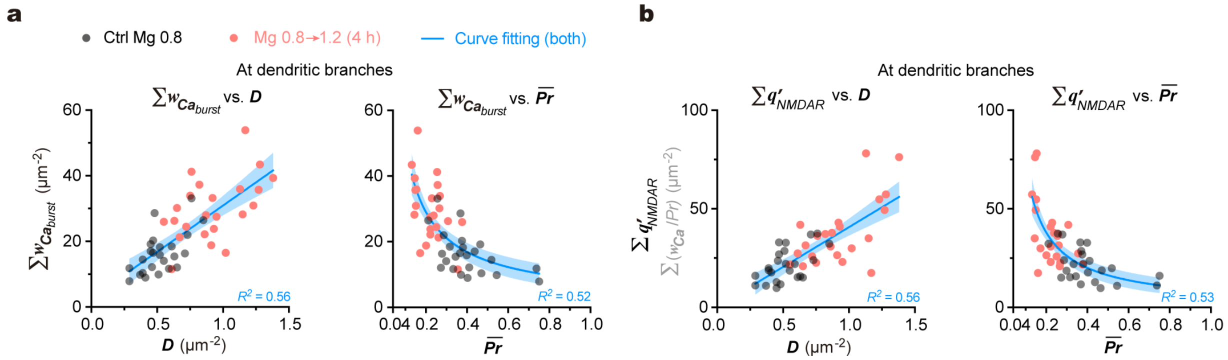

Supplementary Fig. 3 | Properties of postsynaptic NMDAR-conducted Ca^{2+} influx of synapses in dendrites. **a,** Plot of the total $w_{Ca_{burst}}$ ($\Sigma w_{Ca_{burst}}$) per unit area of dendrites against the density (***D***) of synapses (left) or $\overline{Pr}$ (right) (n = 24, 23 branches from 3, 3 repeats, see also **Fig. 3c, f, g**). Linear regression (left, $R^2 = 0.56$), nonlinear regression (right, 8.21•$Pr^{-0.79}$, $R^2 = 0.52$). **b,** Plot of the estimated total q_{NMDAR} ($\Sigma q'_{NMDAR}$) per unit area of dendrites against ***D*** (left) or $\overline{Pr}$ (right). Here, q'_{NMDAR} of individual synapses is equal to w_{Ca}/Pr (n = 24, 23 branches from 3, 3 repeats, see also **Fig. 3i**). Linear regression (left, $R^2 = 0.56$), nonlinear regression (right, 8.59•$Pr^{-0.93}$, $R^2 = 0.53$). Blue lines and error bands, fitted curves and 95% CIs. Source data are provided as a Source Data file.

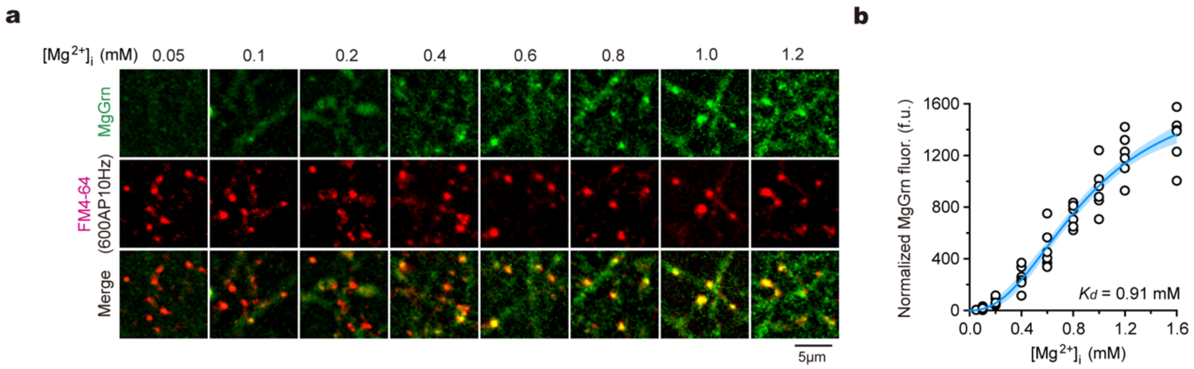

Supplementary Fig. 4 | Calibration of intracellular Mg^{2+} concentrations.

a, Representative confocal images to show MgGrn fluorescence signals with various $[Mg^{2+}]_i$ in boutons. Ionophore was used to equilibrate various concentrations of $[Mg^{2+}]_i$. Following the collection of confocal images of MgGrn, FM4-64 labeling elicited by 600 APs at 10 Hz field simulation was utilized to visualize the boutons and normalize the bouton volume (see **Methods**).

b, Calibration curve (n = 6 biological repeats for each concentration) that was fitted by the Hill equation (R^2 = 0.99, K_d = 0.91 mM). Note the quasi-linear relationship between MgGrn fluorescence and real $[Mg^{2+}]_i$ within the range of 50–1200 f.u. (fluorescence unit). Blue line/error band, fitted curve/95% CI. Source data are provided as a Source Data file.

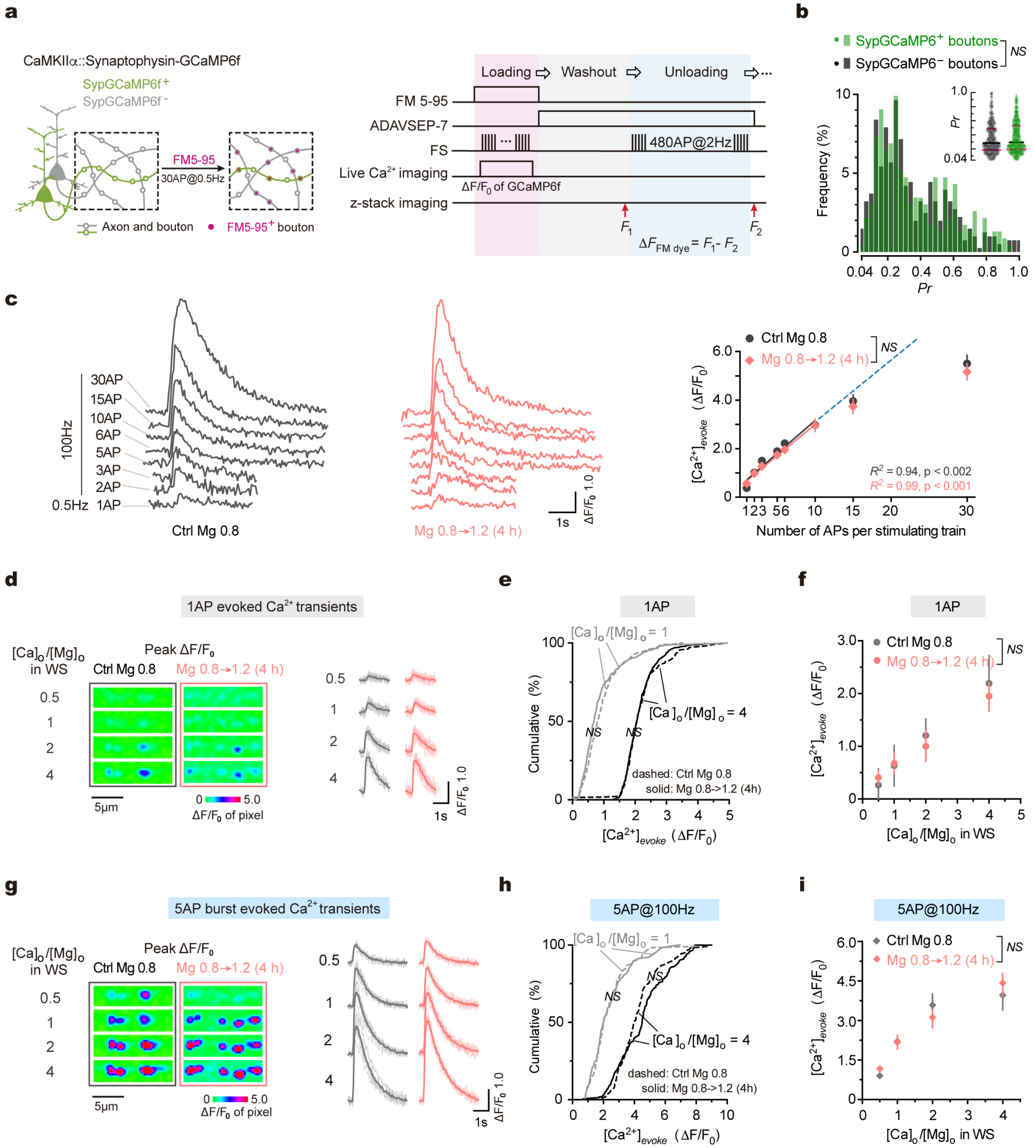

Supplementary Fig. 5 | See next page for caption.

Supplementary Fig. 5 | Measurement of *Pr* and evoked presynaptic Ca^{2+} influx in single boutons

a, Experimental design. Left, schematic to show FM5-95 labeling in the boutons transfected by CaMKIIα-Synaptophysin-GCaMP6f (SypGCaMP6f). Right, Experimental procedures for measuring evoked presynaptic Ca^{2+} influx ($[Ca^{2+}]_{evoke}$) and vesicle turnover (***Pr***) in the same synapses. In loading session, 30AP@0.5Hz or 6 trains of 5AP@100Hz is delivered via field stimulation (FS) to measure ***Pr*** or $\boldsymbol{Pr_{burst}}$. F_1, fluorescence of FM dye loaded in boutons. F_2, residual fluorescence after FM dye unloading. **b, *Pr*** distribution showed no difference in transfected (SypGCaMP6$^+$) and non-transfected (SypGCaMP6$^-$) boutons (n = 406, 232 boutons from 5 repeats). Inset, discrete data points in violin plots, where black and magenta lines indicate median and quartiles. Two-sided Kolmogorov-Smirnov test, P = 0.86 (*NS*). **c,** Left, average traces of Ca^{2+} influx of boutons (visualized by SypGCaMP6f) evoked by various input patterns (n = 302, 387 boutons from 5, 5 repeats). Traces were averaged from 30 sweeps of the boutons. Right, relationship between $[Ca^{2+}]_{evoke}$ and AP number (n = 5 repeats). Solid lines, linear regressions. Dashed line, extension of the black line. The frequency of APs in all bursts was 100 Hz. **d,** Left, representative images of 1AP-evoked peak $\Delta F/F_0$ in the same boutons with various $[Ca^{2+}]_o/[Mg^{2+}]_o$ ratios in working solution (WS) (n = 4, 4 repeats). Right, stacked 30 sweeps of evoked Ca^{2+} influx (thin lines) and their average traces (thick lines). **e,** Cumulative distributions of $[Ca^{2+}]_{evoke}$ of boutons under conditions of $[Ca^{2+}]_o/[Mg^{2+}]_o$ 1 and 4 (n = 217, 253 boutons from 4, 4 repeats). **f,** Plot of average $[Ca^{2+}]_{evoke}$ against $[Ca^{2+}]_o/[Mg^{2+}]_o$ (n = 217, 253 boutons from 4, 4 repeats). **g–i,** The same boutons as in (**d–f**), but with the input of 5AP@100Hz bursts (n = 217, 253 boutons from 4, 4 repeats). In (**g**), stacked 6 sweeps and their average traces were shown. Data are presented as mean ± SEM. Two-sided one-way ANOVA followed by *post hoc* Bonferroni's tests (**c**, **f**, **i**). Two-sided Kolmogorov-Smirnov tests (**c, e, h**). Significance: *NS*, no significance. Source data are provided as a Source Data file.

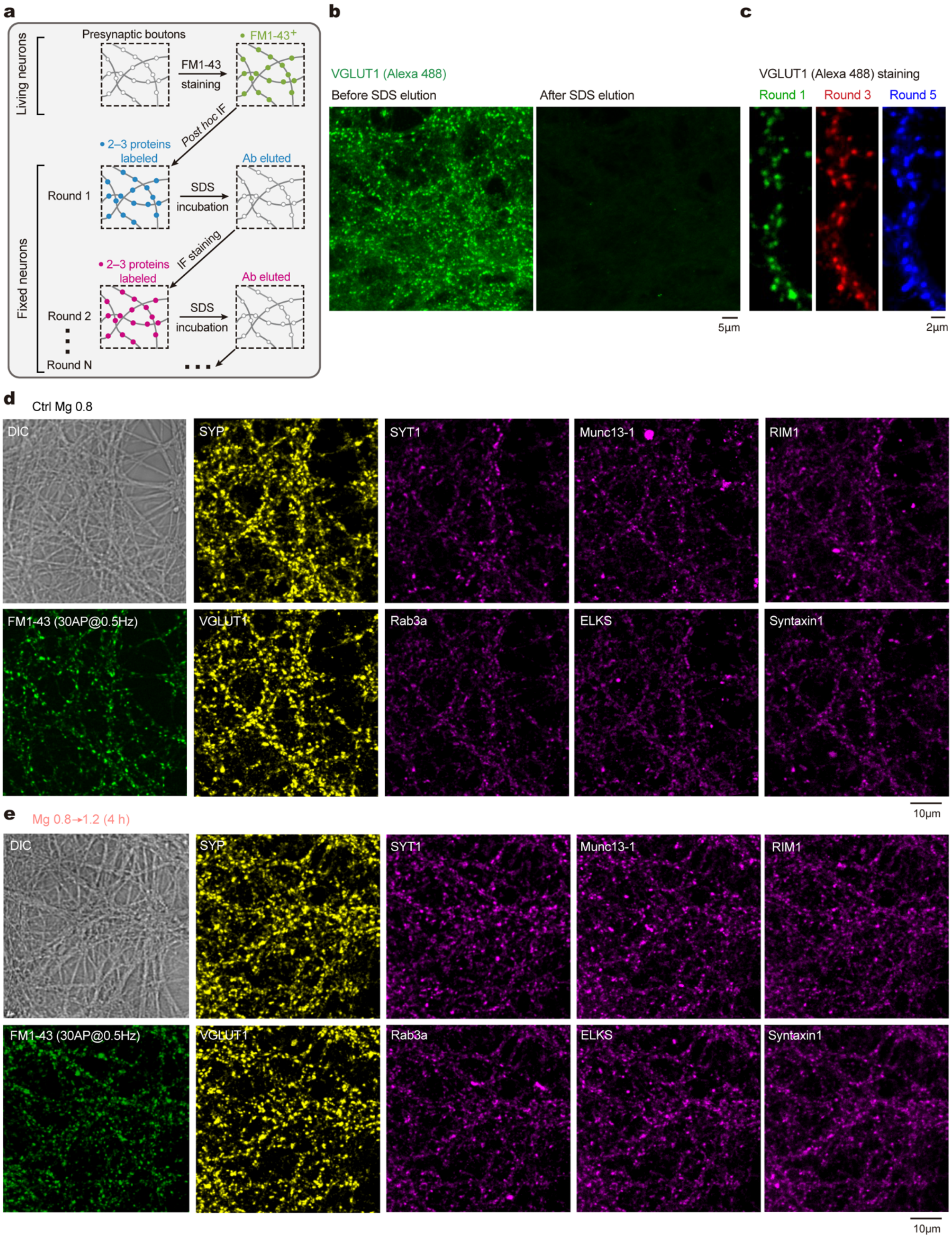

Supplementary Fig. 6 | See next page for caption.

Supplementary Fig. 6 | Labeling of released vesicles and presynaptic proteins in the same boutons.
a, Schematic to show experimental procedures (for details, see **Methods**). Ab, antibody. **b,** Comparison of VGLUT1^{+} immunofluorescence before and after SDS-mediated antibody elution. **c,** Comparison of VGLUT1^{+} immunofluorescence in round 1, 3, and 5 of the staining/eluting cycles. Notably, the VGLUT1 staining is similar after 2 and 4 rounds of elution processes, comparing images from round 3 and 5 with that from round 1). **d, e,** Representative confocal images from physiological (**d**) and elevated Mg^{2+} (**e**) conditions to show the labeling of FM1-43 (30 APs at 0.5 Hz) and *post hoc* immunofluorescence of multiple presynaptic CaSPs in the same region of synaptic network.

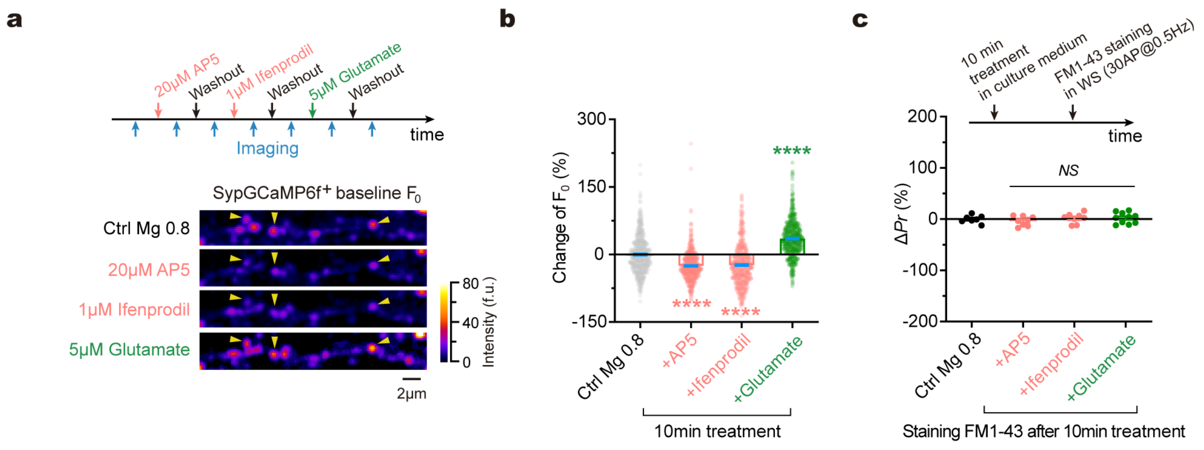

Supplementary Fig. 7 | Acute effects of pharmacological treatments on basal $[Ca^{2+}]_i$ and *Pr* of boutons.

a, Top, experimental procedures. Bottom, changes of baseline fluorescence (F_0) of SypGCaMP6f in the same boutons of an axon 10 min after various pharmacological treatments. **b,** Changes of F_0 (normalized to control, 0%) in the same boutons ($n = 492$ from 6 repeats) 10 min after various treatments. AP5, -25.46 ± 1.53%; ifenprodil, -23.30 ± 2.07%; glutamate, 35.30 ± 1.85%. Two-sided paired *t* tests, ****$P < 0.0001$ for all groups. **c,** Normalized changes in ***Pr*** (**Δ*Pr***) of boutons (normalized to control, 0%) after 10 min treatment of the above drugs ($n = 7, 8, 8, 10$ repeats). Data are shown as mean ± SEM. Two-sided unpaired *t* tests, $P = 0.4376, 0.7172, 0.6467$. *NS*: no significance. Source data are provided as a Source Data file.

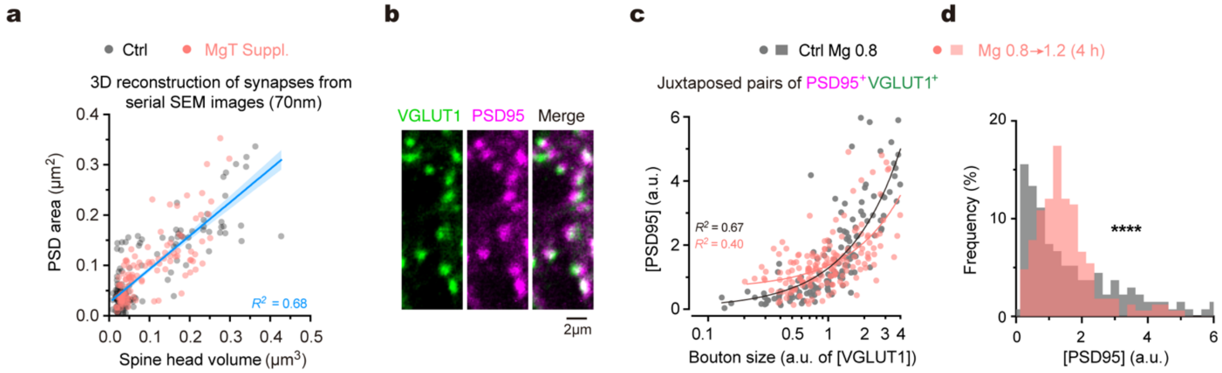

Supplementary Fig. 8 | Elevation of $[Mg^{2+}]_i$ modifies postsynaptic [PSD95] distribution.

a, Plot of PSD area against spine head volume in 3D-reconstructed synapses *in vivo* (*n* = 148, 121 intact spines from 3, 4 rats; linear regression, $R^2 = 0.68$, $P < 0.0001$). Shadow, 95% CI. **b,** Confocal images to show juxtaposed $VGLUT1^+$ and $PSD95^+$ puncta *in vitro*. **c,** Plot of [PSD95] against bouton size (estimated by [VGLUT1]) in single synapses *in vitro* (*n* = 135, 166 synapses from 3, 3 repeats). [PSD95] and [VGLUT1] were median normalized values. Lines, linear regressions, $R^2 = 0.67$, 0.40, $P < 0.0001$ for both. **d,** Distribution of [PSD95] in dendritic spines (data from **c**). Two-sided Kolmogorov-Smirnov test, ****$P < 0.0001$. a.u. in (**c, d**), arbitrary fluorescence unit. Source data are provided as a Source Data file.

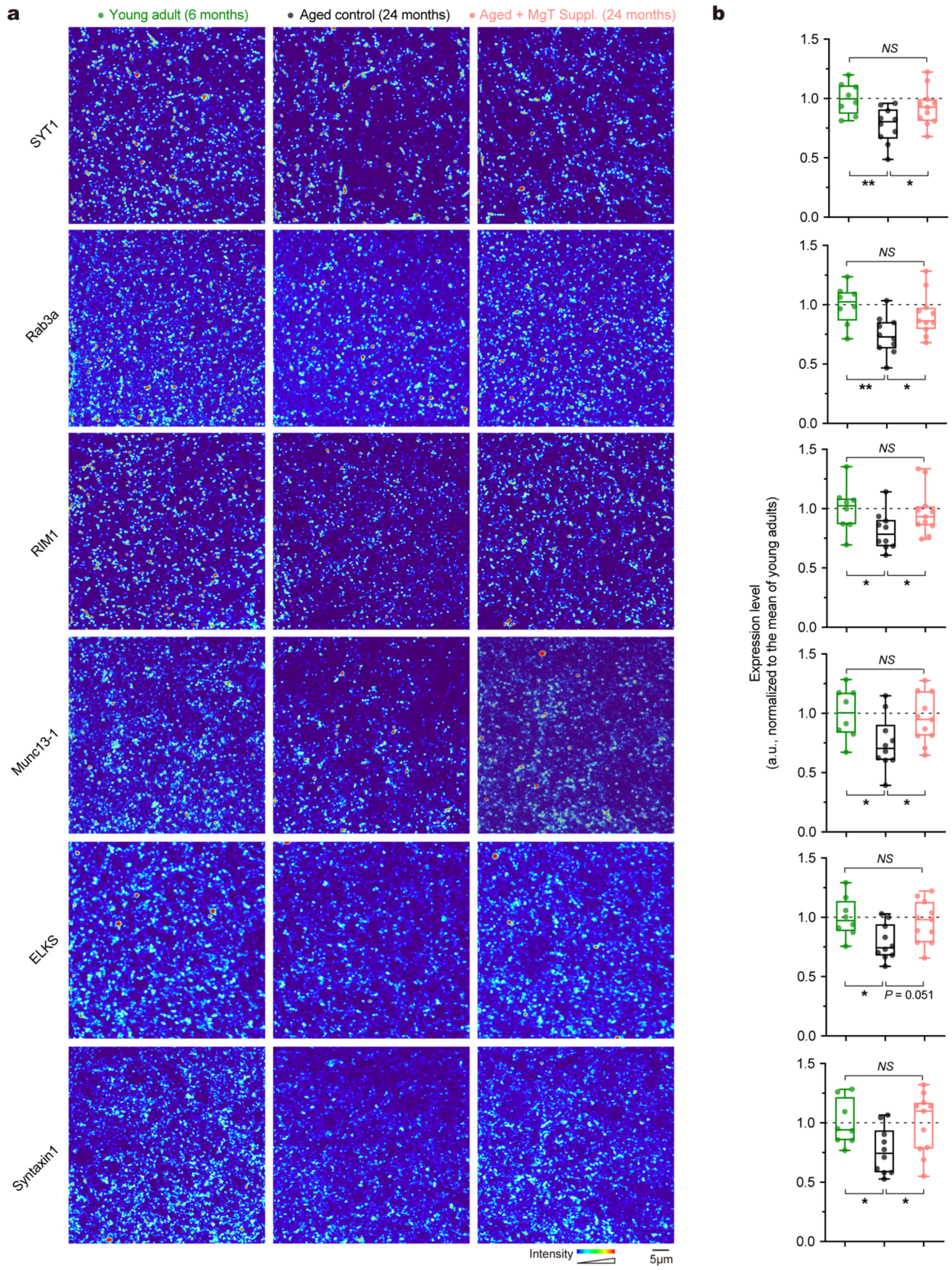

Supplementary Fig. 9 | See next page for caption.

Supplementary Fig. 9 | Brain Mg^{2+} supplementation mitigates aging-induced hippocampal CaSPs decline. **a,** Immunostaining of CaSPs on 70-nm ultrathin slices from the CA1 stratum radiatum (s.r.) region of the hippocampus. From left to right, representative confocal images from young adult rats (6 months of age), aged rats (24 months of age), and aged rats (24 months of age) supplemented with MgT for 8 months (starting from 16 months of age). **b,** Quantification of the protein levels of individual CaSPs (fluorescent intensity of individual rats normalized to the mean of young adults) (n = 8, 10, 11 rats, respectively). By two-sided Mann-Whitney tests, SYT1: P = 0.0062, 0.0430, 0.2723; Rab3a: P = 0.0085, 0.0295, 0.2723; RIM1: P = 0.0434, 0.0357, 0.4920; Munc13-1: P = 0.0205, 0.0357, 0.7168; ELKS: P = 0.0205, 0.0513, 0.7168; Syntaxin1: P = 0.0266, 0.0357, > 0.9999. Source data are provided as a Source Data file.

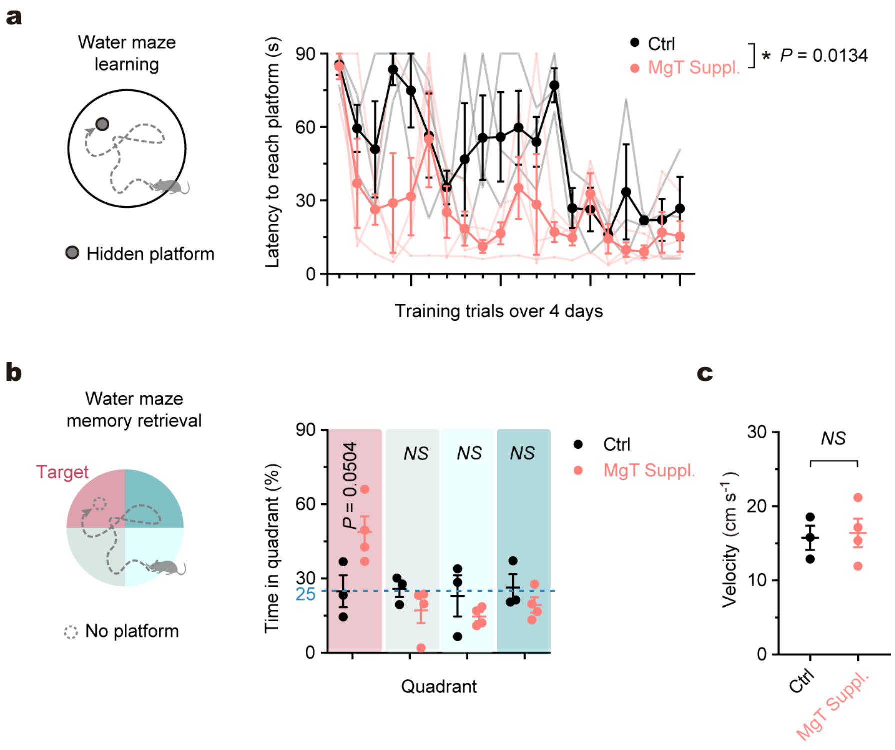

Supplementary Fig. 10 | Elevating brain Mg^{2+} levels improves learning and memory in aged animals.
a, Water maze learning curves throughout training sessions (n = 3, 4 rats for Ctrl and MgT suppl. groups at 26 months of age, from the same animals in **Fig. 7**). Transparent curves represent individual animals. Two-sided two-way ANOVA followed by *post hoc* Bonferroni's test, *$P = 0.0134$, $F_{(5, 95)} = 3.0535$. **b,** Time spent in quadrants in the testing session 3 days after the last training trial (P = 0.0504, 0.2596, 0.2919, 0.3111). Dashed line, chance level. **c,** Swimming velocity of individual animals (P = 0.8128). Two-sided unpaired t tests (**b, c**). *NS*, no significance. Data are shown as mean ± SEM. Source data are provided as a Source Data file.

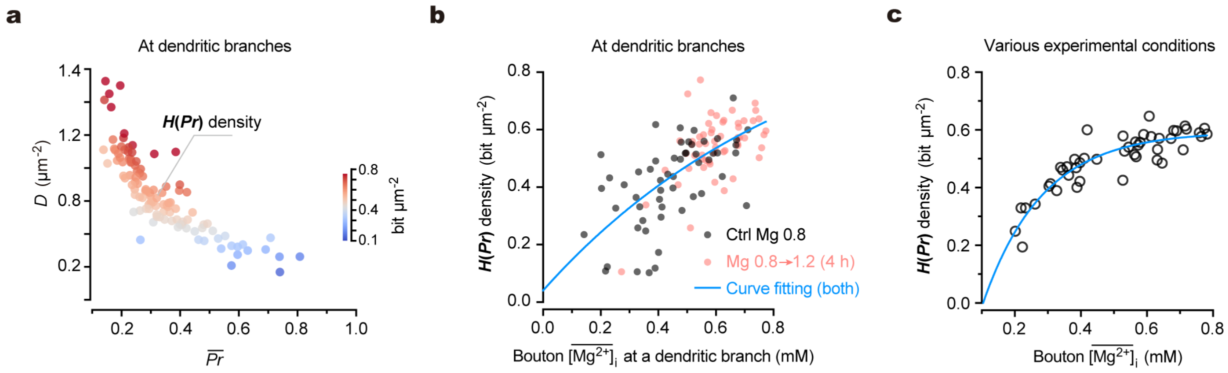

Supplementary Fig. 11 | Synaptic configuration sets coding capacity of synapses at dendrites.

a, Plot of synaptic configurations and their corresponding density of synaptic information entropy ***H*(*Pr*)**, defined by the entropy per unit area of dendrites (bit μm^{-2}, coded by pseudo color) at individual dendritic branches (n = 58, 53 branches from 4, 4 repeats; data from **Fig. 1e**). Notably, branches with $\boldsymbol{D^{Hi}\,\overline{Pr}^{\,Lo}}$ configuration have higher ***H*(*Pr*)** density. **b,** Plot of ***H*(*Pr*)** density against mean bouton $[Mg^{2+}]_i$ at individual dendritic branches (n = 55, 56 branches from 4, 4 repeats; data from **Fig. 4e, 5a**). Blue curve, one-phase association (R^2 = 0.45). **c,** Similar to (**b**), but the data were from **Fig. 6e** (n = 46 various experimental conditions). Each data point represents the average value under each condition. Blue curve, one-phase association (R^2 = 0.82). Source data are provided as a Source Data file.

Supplementary Table 1 | Biophysical variables.

Variable	Description	Experiments	Approximation	Figures
**Single synapses**				
$\boldsymbol{Pr}$	Vesicle release probability in basal transmission	FM staining	No.	1c, d; 2a, c; 3a, i, j; 4d; 5c, f; S5b
$\boldsymbol{Pr_{burst}}$	Vesicle release probability in bursting transmission	FM staining	No.	1d
$\boldsymbol{q}$	Quantal size of excitatory synaptic transmission (via AMPAR)	GluA2, PSD95 IF	$\boldsymbol{q'}$ = [GluA2] or [PSD95]	2a, c; S2a
$\boldsymbol{w}$	Excitatory synaptic weight in basal transmission (via AMPAR)	GluA2, PSD95 IF	$\boldsymbol{w'} = \boldsymbol{Pr}$•[GluA2] or $\boldsymbol{Pr}$•[PSD95]	S2a
$\boldsymbol{w_{burst}}$	Excitatory synaptic weight in bursting transmission (via AMPAR)	GluA2, PSD95 IF	$\boldsymbol{w'_{burst}} = \boldsymbol{Pr_{burst}}$•[GluA2] or $\boldsymbol{Pr_{burst}}$•[PSD95]	
$\boldsymbol{q_{NMDAR}}$	Quantal release-induced NMDAR current (*i.e.*, upon 1 released vesicle)	SypGCaMP6f imaging; GluN2B transfection	$\boldsymbol{q'_{NMDAR}} = \boldsymbol{w_{Ca}}/\boldsymbol{Pr}$ or $[GluN2B]_{Sp}$	3i, j
$\boldsymbol{w_{Ca}}$	Spine Ca^{2+} entry in basal transmission (1AP-evoked)	SypGCaMP6f imaging	No.	3a, d, e
$\boldsymbol{w_{Ca_{burst}}}$	Spine Ca^{2+} entry in bursting transmission (burst-evoked)	SypGCaMP6f imaging	No.	3e
**Dendritic branches**				
$\boldsymbol{D}$	Functional synapse density at dendritic branches	FM staining	No.	1b, e; 2b, d, f; 3c, f; 4a, e; 5a; 6a, b, c, e; S1d; S2c; S3; S11a
$\overline{\boldsymbol{Pr}}$	Average $\boldsymbol{Pr}$ of functional synapses at dendritic branches	FM staining	No.	1b, e; 4a, e; 6a, b, c, e; S1c; S2d; S3; S7c; S11a
$\Sigma\boldsymbol{Pr}$	Total presynaptic weight per unit area of dendrites in basal transmission	FM staining	No. Equal to $\boldsymbol{D}$•$\overline{\boldsymbol{Pr}}$	1b; S1e
$\Sigma\boldsymbol{Pr_{burst}}$	Total presynaptic weight per unit area of dendrites in bursting transmission	FM staining	No.	1f; S1e;
$\bar{\boldsymbol{q}}$	Average $\boldsymbol{q}$ of synapses at dendritic branches	GluA2, PSD95 IF	$\overline{\boldsymbol{q'}} = \overline{[GluA2]}$ or $\overline{[PSD95]}$	2b, d, f; S2b, e
$\boldsymbol{G}$	Total excitatory synaptic weight per unit area of dendrites in basal transmission (or the basal *Gain*)	FM staining; GluA2, PSD95 IF	$\boldsymbol{G'} = \Sigma\boldsymbol{w'}$/dendritic area	2f; S2b-e;
$\boldsymbol{G_{burst}}$	Total excitatory synaptic weight per unit area of dendrites in bursting transmission (or the bursting *Gain*)	FM staining; GluA2, PSD95 IF	$\boldsymbol{G'_{burst}} = \Sigma\boldsymbol{w'_{burst}}$/dendritic area	2f
$\overline{\boldsymbol{w_{Ca}}}$	Average spine Ca^{2+} entry in basal transmission at dendritic branches	FM staining	No.	3c, f
$\Sigma\boldsymbol{w_{Ca}}$	Total spine Ca^{2+} entry per unit area of dendrites in basal transmission (1AP-evoked)	Spine GCaMP6f imaging	No.	3f, g
$\Sigma\boldsymbol{w_{Ca_{burst}}}$	Total spine Ca^{2+} entry per unit area of dendrites in bursting transmission (burst-evoked)	Spine GCaMP6f imaging	No.	3f, g; S3b
$\boldsymbol{H(Pr)}$	Information entropy of a bouton	FM staining	No.	S11

Note: IF, immunofluorescence

Supplementary Table 2 | Reagents and resource.

REAGENT or RESOURCE	SOURCE	IDENTIFIER
**Antibodies**		
Rabbit polyclonal anti-ERC1b/2 (ELKS)	Synaptic Systems	Cat#143003; RRID: AB_887715
Mouse monoclonal anti-GluA2 (clone 6C4)	Invitrogen	Cat#32-0300; RRID: AB_2533058
Guinea pig polyclonal anti-MAP2	Synaptic Systems	Cat#188 004; RRID: AB_2138181
Mouse monoclonal anti-Munc13-1 (clone 266B1)	Synaptic Systems	Cat#126 111; RRID: AB_887735
Rabbit polyclonal anti-Munc13-1	Synaptic Systems	Cat#126103; RRID: AB_887733
Mouse monoclonal anti-PSD95 (clone 7E3-1B8)	Millipore	Cat#CP35; RRID: AB_2092542
Mouse monoclonal anti-Rab3a (clone 42.2)	Synaptic Systems	Cat#107111; RRID: AB_887770
Rabbit polyclonal anti-Rab3a	Synaptic Systems	Cat#107102; RRID: AB_887769
Rabbit polyclonal anti-RIM1	Synaptic Systems	Cat#140003; RRID: AB_887774
Mouse monoclonal anti-Synaptophysin (clone SY38)	Millipore	Cat#MAB5258; RRID: AB_2313839
Guinea pig polyclonal anti-Synaptophysin	Synaptic Systems	Cat#101004; RRID: AB_1210382
Mouse monoclonal anti-Synaptotagmin1 (clone 41.1)	Synaptic Systems	Cat#105011; RRID: AB_887832
Mouse monoclonal anti-Syntaxin1 (clone 78.2)	Synaptic Systems	Cat#110011; RRID: AB_887844
Guinea pig polyclonal anti-VGLUT1	Millipore	Cat#AB5905; RRID: AB_2301751
CF488A Goat Anti-Guinea pig IgG (H+L)	Biotium	Cat#20017; RRID: AB_10559033
CF488A Goat Anti-Mouse IgG (H+L)	Biotium	Cat#20018; RRID: AB_10557263
CF488A Goat Anti-Rabbit IgG (H+L)	Biotium	Cat#20019; RRID: AB_10583180
CF555 Goat Anti-Guinea pig IgG (H+L)	Biotium	Cat#20036; RRID: AB_10557404
CF555 Goat Anti-Mouse IgG (H+L)	Biotium	Cat#20231; RRID: AB_10854844
CF555 Goat Anti-Rabbit IgG (H+L)	Biotium	Cat#20232; RRID: AB_10871474
CF640R Goat Anti-Guinea pig IgG (H+L)	Biotium	Cat#20085; RRID: AB_10853612
CF640R Goat Anti-Mouse IgG (H+L)	Biotium	Cat#20175; RRID: AB_10853622
CF640R Goat Anti-Rabbit IgG (H+L)	Biotium	Cat#20176; RRID: AB_10854992
**Chemicals, Peptides, and Recombinant Proteins**		
8-Bromoadenosine 3′,5′-cyclic monophosphate (8-Br-cAMP)	Sigma-Aldrich	Cat#B5386; Cas#23583-48-4
ADVASEP-7	Biotium	Cat#70029
Bovine Serum Albumin (BSA)	Amresco	Cat#0332
Brain-derived neurotrophic factor (BDNF)	Sigma-Aldrich	Cat#B3795
Chloral hydrate	Sigma-Aldrich	Cat#C8383; Cas#302-17-0
DL-2-Amino-5-phosphonopentanoic acid (DL-AP5)	Sigma-Aldrich	Cat#A5282; Cas#76326-31-3
Ifenprodil (+)-tartrate salt	Sigma-Aldrich	Cat#I2892; Cas#23210-58-4
Imipramine hydrochloride	Sigma-Aldrich	Cat#I7379; Cas#113-52-0
Kynurenic Acid	Sigma-Aldrich	Cat#K3375; Cas#492-27-3
L-Glutamic acid monosodium salt	Sigma-Aldrich	Cat#49621; Cas#6106-04-3
LR White resin	E.M.S.	Cat#14380-14382
Magnesium Green AM ester	Molecular Probes	Cat#M3735
Magnesium L-threonate (MgT)	NeuroCentria Inc.	Cat#L-TAMS
NBQX disodium salt hydrate	Sigma-Aldrich	Cat#N183; Cas#118876-58-7
$PKI_{14\text{-}22}$ Amide	Tocris	Cat#2546; Cas#201422-03-9
Recombinant Human sTNF RI/TNFRSF1A Protein (sTNFR1)	R&D systems	Cat#636-R1-025

(Supplementary Table 2 Continued)

Recombinant Human TNF-alpha Protein (TNF-α)	R&D systems	Cat#210-TA-100
Ro25-6981	Tocris	Cat#1594; Cas#1312991-76-6
Saponin	Sigma-Aldrich	Cat#47036; Cas#8047-15-2
SPI-Pon 812 Embedding Kit (Epoxy resin)	SPI-CHEM	Cat#02660-AB
SynaptoGreen C4 (FM1-43)	Biotium	Cat#70020; Cas#149838-22-2
SynaptoRed C2 (FM4-64)	Biotium	Cat#70021
SynaptoRed C2M (FM5-95)	Biotium	Cat#70028
Tetrodotoxin (TTX)	Sigma-Aldrich	Cat#T8024; Cas#4368-28-9
CalPhos Mammalian Transfection Kit	Clontech	Cat#631312
**Recombinant DNA**		
Plasmid: CaMKIIα-Synaptophysin-GCaMP6f	G.-Q.B.'s lab	N.A.
Plasmid: CaMKIIα-GCaMP6f	G.-Q.B.'s lab	N.A.
Plasmid: CaMKIIα-GFP-NR2B	Luo *et al.*	N.A.
Plasmid: CaMKIIα-mKate2	G.-Q.B.'s lab	N.A.

Supplementary Notes

- **Serial studies regarding the role of Mg^{2+} on brain health and aging**

As this study is part of a series investigating the positive impact of Mg^{2+} on brain health and aging, we would like to provide a concise overview of our serial studies, which explore the role of Mg^{2+} ions in synaptic, neuronal, circuitry, and cognitive functions over the years. Our investigations span from *in vitro* experiments to *in vivo* studies involving animals and humans, from the micro-level of proteins and single synapses to the macro-level behaviors and cognitive functions.

Initially, we observed that elevating extracellular Mg^{2+} concentration enhanced long-term potentiation (LTP) of synapses in cultured hippocampal neurons, leading to increased expression of GluN2B-containing NMDARs [1]. Building upon this discovery, we hypothesized that raising brain Mg^{2+} levels could improve synaptic plasticity in the hippocampus, thereby enhancing cognitive functions, especially learning and memory, in intact animals. To achieve this, we developed Magnesium L-Threonate (MgT), a compound that effectively increased Mg^{2+} bioavailability in the cerebrospinal fluid (CSF) when orally consumed [2]. Elevating Mg^{2+} in the rodent brain's CSF demonstrated enhanced synaptic plasticity and cognitive functions in both young and aging animals [2], validating our *in vitro* hypotheses. Concurrently, we observed beneficial effects in treating cognitive declines in Alzheimer's disease model mice [3] and depression model mice [4].

Encouraged by these animal studies, we expanded our research to translational studies. The first double-blind placebo-controlled clinical study demonstrated that MgT supplementation improves cognitive functions in mild cognitive impairment (MCI) patients [5]. Currently, three ongoing FDA-approved phase 2b/3 clinical trials are investigating MgT's role in treating cognitive disorders in humans, including Alzheimer's disease [6], Attention Deficit Hyperactivity Disorder (ADHD) [7], and depression/anxiety.

Despite these promising clinical studies, the mechanism underlying the powerful impact of Mg^{2+} on human brain functions remains elusive. Initially, we believed that the primary effect of extracellular Mg^{2+} targets NMDARs to influence plasticity based on electrophysiological and molecular evidence [1], demonstrating its extracellular modulatory effect. However, we later discovered that the beneficial effects extend beyond modulating synaptic plasticity. Subsequently, our findings revealed that intracellular Mg^{2+} plays an even more crucial role in regulating the density of functional presynaptic boutons [8], offering a new perspective on Mg^{2+}'s role in promoting brain health.

Intriguingly, in the compound MgT, threonate (T) itself synergizes with Mg^{2+}, elevating intracellular Mg^{2+} levels and increasing the density of presynaptic boutons in cultured hippocampal neurons [9]. This insight contributes to understanding the pharmacological effects of MgT in elevating brain Mg^{2+} levels and enhancing animal cognitive functions. Despite focusing on single synapses in these mechanistic studies, it remains unclear how intracellular Mg^{2+} governs multiple synapses along individual dendritic branches, imparting different transmission efficiency, plasticity, and coding capacity. Given the fundamental role of dendritic branches in processing information, addressing this question could illuminate how nearby synapses are regulated to achieve specific computational features at individual dendritic branches and identify endogenous factors controlling such synaptic organization.

• The concept of synaptic configuration

In the current article, the landing point is to tackle a longstanding question in the field: how nearby synapses at individual dendritic branches are organized to generate distinct synaptic computations, essentially regulating the "transfer function" of synapses at a dendritic branch. This question is crucial as dendritic branches are considered the basic computational unit for information processing underlying cognitive functions. Our findings reveal that intracellular Mg^{2+} serves as an endogenous factor in organizing nearby synapses from different presynaptic neurons, influencing the configuration of synaptic connectivity at individual dendritic branches. This, in turn, determines the "transfer function" of each dendritic branch. We introduced a general principle of synaptic organization at dendritic branches, proposing that nearby synapses are consistently organized along an individual branch to maintain a constant total presynaptic strength (the first part of the Discussion).

It's important to note that the concept of *configuration* is more generalized, with the regulatory effect of intracellular Mg^{2+} serving as a significant example. As different configurations impart distinct features of synaptic computations to an individual branch, the transition between configurations becomes crucial for branch-specific synaptic computations during information processing for learning and memory. Significantly, our principle hints at the possibility of other essential endogenous factors, beyond intracellular Mg^{2+}, regulating synaptic configuration. Such factors could be promising candidates for anti-brain aging and anti-neurodegeneration strategies, providing a novel avenue for drug exploration. Overall, we believe that this study offers precise and comprehensive mechanisms, serving as a cornerstone in our series of studies on the beneficial effects of brain Mg^{2+} in maintaining brain health.

• Rationales for the experimental Mg^{2+} condition

Mg^{2+} stands as the second most abundant intracellular mineral after K^+ and is present in substantial amounts in the cerebrospinal fluid (CSF) of both rodents (around 0.8 mM) and humans (around 1.0–1.2 mM in healthy individuals) (for a review [10]). The concentrations of 0.8–1.2 mM used in the current study are supported by multiple lines of evidence. Under *in vivo* conditions, $[Mg^{2+}]_o$ in the CSF of animal brains can increase by 21% above control (*i.e.*, from ~1 mM to 1.2 mM) 5.5 hours after intravenous injection of $MgCl_2$ or $MgSO_4$ (Ref [11]). Similarly, $[Mg^{2+}]_o$ in the CSF of human brains can be raised from 0.95 ± 0.11 to 1.13 ± 0.19 mM by intravenous injection of $MgSO_4$ (Ref [12]). In our studies, we demonstrated in living rats that oral MgT treatment can elevate $[Mg^{2+}]_o$ in the CSF by 15% (~0.2 mM) through water consumption [2]. Other studies in living mice, using advanced techniques to measure brain interstitial $[Mg^{2+}]_o$, reported that during the transition from wakefulness to sleep, $[Mg^{2+}]_o$ quickly increases by ~0.13 mM from a baseline of ~0.7 mM; conversely, during the transition from sleep to wakefulness, $[Mg^{2+}]_o$ decreases by ~0.11 mM from a baseline of ~1 mM (Ref [13]). Importantly, they demonstrated variations in $[Mg^{2+}]_o$ among individual animals, ranging from ~0.5–1.2 mM (Ref [13]), indicating that $[Mg^{2+}]_o$ can vary by up to twofold in mouse brains. Additionally, during the transition from wakefulness to isoflurane anesthesia in mice, brain $[Mg^{2+}]_o$ can increase by ~0.44 mM (ranging from ~0.5–1.5 mM in different mice), illustrating a notable brain state-dependent change in $[Mg^{2+}]_o$ (Ref [13]).

Therefore, the concentrations of $[Mg^{2+}]_o$ employed in our *in vitro* model system, 0.8–1.2 mM, fall

within the physiologically relevant range observed under *in vivo* conditions.

- **Aging is a risk for Mg^{2+} deficits**

Aging poses a significant risk for Mg^{2+} deficit, as highlighted in various reviews [10, 14-21]. Clinical studies reveal a substantial decrease in brain cerebrospinal fluid (CSF) Mg^{2+} concentration during aging and neurodegenerative diseases in humans [22]. Notably, elemental Mg^{2+} levels are markedly reduced in the brains of Alzheimer's disease patients [23, 24]. As regard to intracellular Mg^{2+} levels, clinical studies employed the phosphorus magnetic resonance spectrum (^{31}P MRS), a method for measuring intracellular ionized Mg^{2+} concentrations *in vivo*, demonstrate a significant decrease in body $[Mg^{2+}]_i$ during aging [25, 26]. These findings suggest that the decline in $[Mg^{2+}]_i$ serves as a hallmark of aging and neurodegeneration, emphasizing the crucial role of Mg^{2+} in protecting brain health. Indeed, both animal and human studies underscore the effectiveness of brain Mg^{2+} supplementation in addressing cognitive deficits associated with aging and neurodegenerative disorders.

In animal studies, brain Mg^{2+} supplementation exhibits a protective effect against aging-dependent cognitive declines [10, 27]. Our research demonstrates that cognitive impairments in aged animals [2] and Alzheimer's disease model animals [3] can be significantly ameliorated through brain Mg^{2+} supplementation. Additionally, brain Mg^{2+} supplementation shows promise in treating other neurodegenerative diseases. Independent studies report that MgT treatment effectively alleviates motor deficits and dopamine neuron loss in a mouse model of Parkinson's disease [28].

Translational research assesses the efficacy of MgT (also known as L-threonic acid magnesium salt, L-TAMS) treatment in ameliorating cognitive deficits related to aging and neurological disorders. In our initial double-blind, placebo-controlled clinical study, MgT supplementation is shown to significantly reverse age-dependent cognitive impairment [5]. Consistent results are reproduced in other double-blind, placebo-controlled clinical studies conducted by independent groups [29]. Moreover, a clinical trial by Stanford University researchers demonstrates that MgT treatment effectively alleviates cognitive decline in Alzheimer's disease patients [30]. Another open-label pilot study at Massachusetts General Hospital reports that MgT treatment improves cognitive functions in ADHD patients [7].

Recently, the World Health Organization reached a consensus that dietary Mg^{2+} intake is lower than recommended in a majority of the world's population, especially in the aging demographic (https://www.who.int/publications/i/item/9789241563550; see also clinical trials [31, 32]). Therefore, based on the compelling evidence, elevating brain Mg^{2+} levels in the elderly emerges as a promising strategy to minimize, or even prevent, aging-dependent cognitive deficits.

- **Implications of Mg^{2+} deficits for brain aging**

Over the past decades, numerous animal and clinical studies have extensively documented progressive deficits in body Mg^{2+} levels during aging, likely stemming from insufficient intake and disorders in Mg^{2+} metabolism (for reviews [14-20]). However, the underlying mechanisms still require in-depth exploration. Mg^{2+} deficiency emerges as a high-risk factor for brain aging and neurodegeneration,

crucial for sustaining brain health in both young and aged animals.

Firstly, Mg^{2+} sufficiency proves pivotal for maintaining brain health in young adults. On one hand, a 30–35% reduction in dietary Mg^{2+} causes a 40% decrease in $[Mg^{2+}]_i$ in the brains of young adult animals [33], leading to significant impairments in cognitive functions, especially hippocampus-dependent learning and memory (for examples see Refs [34-36]). Moreover, dietary Mg^{2+} deficiency induces systemic low-grade neuroinflammation in young adults, a hallmark of aging and neurodegenerative diseases (for a review [37]). On the other hand, an early study reported that chronic feeding of a high-Mg^{2+} diet (2% elemental Mg^{2+} in the diet) increases brain Mg^{2+} levels and improves learning behaviors in young rats [38]. Consistently, our studies have demonstrated that when young animals consume a normal-Mg^{2+} diet, supplementation of brain Mg^{2+} through oral intake of MgT in drinking water further enhances their learning and memory [2].

Secondly, Mg^{2+} supplementation reverses cognitive declines in aging and neurodegeneration. Early studies have reported an improvement in cognitive functions in aged animals through a high dosage of Mg^{2+} in the diet [38]. Our previous studies show restored learning and memory in aged rats by elevating brain Mg^{2+} levels through MgT treatment [2]. Additionally, we demonstrate that cognitive declines can be effectively ameliorated by MgT treatment in Alzheimer's disease model mice (APP/PS1 transgenic mice) [3]. Consistently, an independent study indicates that MgT treatment can reduce neuroinflammation and alleviate cognitive decline in APP/PS1 transgenic mice [39].

Overall, converging evidence suggests a crucial role of Mg^{2+} in maintaining brain health in young adults and during brain aging.

References

1. Slutsky, I., Sadeghpour, S., Li, B. & Liu, G. Enhancement of synaptic plasticity through chronically reduced Ca^{2+} flux during uncorrelated activity. *Neuron* **44**, 835-849 (2004).
2. Slutsky, I., *et al.* Enhancement of learning and memory by elevating brain magnesium. *Neuron* **65**, 165-177 (2010).
3. Li, W., *et al.* Elevation of brain magnesium prevents synaptic loss and reverses cognitive deficits in Alzheimer's disease mouse model. *Mol Brain* **7**, 65 (2014).
4. Abumaria, N., *et al.* Effects of elevation of brain magnesium on fear conditioning, fear extinction, and synaptic plasticity in the infralimbic prefrontal cortex and lateral amygdala. *J Neurosci* **31**, 14871-14881 (2011).
5. Liu, G., Weinger, J.G., Lu, Z.L., Xue, F. & Sadeghpour, S. Efficacy and Safety of MMFS-01, a Synapse Density Enhancer, for Treating Cognitive Impairment in Older Adults: A Randomized, Double-Blind, Placebo-Controlled Trial. *J Alzheimers Dis* **49**, 971-990 (2016).
6. Weinger, J.G. & Liu, G. [P4–001]: MMFS TREATMENT AMELIORATES FRONTAL CORTEX DYSFUNCTION IN MILD-MODERATE ALZHEIMER's DISEASE PATIENTS. *Alzheimer's & Dementia* **13**, P1253-P1253 (2017).
7. Surman, C., *et al.* L-Threonic Acid Magnesium Salt Supplementation in ADHD: An Open-Label Pilot Study. *J Diet Suppl* **18**, 119-131 (2021).
8. Zhou, H. & Liu, G. Regulation of density of functional presynaptic terminals by local energy supply. *Mol Brain* **8**, 42 (2015).
9. Sun, Q., Weinger, J.G., Mao, F. & Liu, G. Regulation of structural and functional synapse density by L-threonate through modulation of intraneuronal magnesium concentration. *Neuropharmacology* **108**, 426-439 (2016).
10. Billard, J.M. Brain free magnesium homeostasis as a target for reducing cognitive aging. in *Magnesium in the Central Nervous System* (ed. R. Vink & M. Nechifor) (Adelaide (AU), 2011).
11. Oppelt, W.W., MacIntyre, I. & Rall, D.P. Magnesium exchange between blood and cerebrospinal fluid. *Am J Physiol* **205**, 959-962 (1963).
12. Fuchs-Buder, T., Tramer, M.R. & Tassonyi, E. Cerebrospinal fluid passage of intravenous magnesium sulfate in neurosurgical patients. *J Neurosurg Anesthesiol* **9**, 324-328 (1997).
13. Ding, F., *et al.* Changes in the composition of brain interstitial ions control the sleep-wake cycle. *Science* **352**, 550-555 (2016).
14. Barbagallo, M., Veronese, N. & Dominguez, L.J. Magnesium in Aging, Health and Diseases. *Nutrients* **13** (2021).
15. Barbagallo, M. & Dominguez, L.J. Magnesium and aging. *Curr Pharm Des* **16**, 832-839 (2010).

16. Durlach, J., *et al.* Magnesium status and ageing: an update. *Magnes Res* **11**, 25-42 (1998).
17. Durlach, J., *et al.* Magnesium and ageing. II. Clinical data: aetiological mechanisms and pathophysiological consequences of magnesium deficit in the elderly. *Magnes Res* **6**, 379-394 (1993).
18. Rayssiguier, Y., Durlach, J., Gueux, E., Rock, E. & Mazur, A. Magnesium and ageing. I. Experimental data: importance of oxidative damage. *Magnes Res* **6**, 369-378 (1993).
19. Barbagallo, M., Belvedere, M. & Dominguez, L.J. Magnesium homeostasis and aging. *Magnes Res* **22**, 235-246 (2009).
20. Durlach, J., *et al.* Are age-related neurodegenerative diseases linked with various types of magnesium depletion? *Magnes Res* **10**, 339-353 (1997).
21. Billard, J.M. Ageing, hippocampal synaptic activity and magnesium. *Magnes Res* **19**, 199-215 (2006).
22. Basun, H., Forssell, L.G., Wetterberg, L. & Winblad, B. Metals and trace elements in plasma and cerebrospinal fluid in normal aging and Alzheimer's disease. *J Neural Transm Park Dis Dement Sect* **3**, 231-258 (1991).
23. Andrasi, E., Igaz, S., Molnar, Z. & Mako, S. Disturbances of magnesium concentrations in various brain areas in Alzheimer's disease. *Magnes Res* **13**, 189-196 (2000).
24. Andrasi, E., Pali, N., Molnar, Z. & Kosel, S. Brain aluminum, magnesium and phosphorus contents of control and Alzheimer-diseased patients. *J Alzheimers Dis* **7**, 273-284 (2005).
25. Binzoni, T., *et al.* Age dependence of human gastrocnemius Mg^{2+}: fitting ^{31}P-NMR spectra using quantum mechanics-based prior knowledge. *J Physiol Anthropol Appl Human Sci* **20**, 275-283 (2001).
26. Cameron, D., *et al.* Age and Muscle Function Are More Closely Associated With Intracellular Magnesium, as Assessed by ^{31}P Magnetic Resonance Spectroscopy, Than With Serum Magnesium. *Front Physiol* **10**, 1454 (2019).
27. Toffa, D.H., Magnerou, M.A., Kassab, A., Hassane Djibo, F. & Sow, A.D. Can magnesium reduce central neurodegeneration in Alzheimer's disease? Basic evidences and research needs. *Neurochem Int* **126**, 195-202 (2019).
28. Shen, Y., *et al.* Treatment Of Magnesium-L-Threonate Elevates The Magnesium Level In The Cerebrospinal Fluid And Attenuates Motor Deficits And Dopamine Neuron Loss In A Mouse Model Of Parkinson's disease. *Neuropsychiatr Dis Treat* **15**, 3143-3153 (2019).
29. Zhang, C., *et al.* A Magtein((R)), Magnesium L-Threonate, -Based Formula Improves Brain Cognitive Functions in Healthy Chinese Adults. *Nutrients* **14** (2022).
30. Wroolie, T.E., *et al.* OPEN LABEL TRIAL OF MAGNESIUM L-THREONATE IN PATIENTS WITH DEMENTIA. *Innovation in Aging* **1**, 170-170 (2017).
31. Ford, E.S. & Mokdad, A.H. Dietary magnesium intake in a national sample of US adults. *J Nutr* **133**, 2879-2882 (2003).
32. Galan, P., *et al.* Dietary magnesium intake in a French adult population. *Magnes Res* **10**, 321-328 (1997).
33. Altura, B.M., Gebrewold, A., Zhang, A., Altura, B.T. & Gupta, R.K. Short-term reduction in dietary intake of magnesium causes deficits in brain intracellular free Mg^{2+} and H^+ but not high-energy phosphates as observed by in vivo ^{31}P-NMR. *Biochim Biophys Acta* **1358**, 1-5 (1997).
34. Bardgett, M.E., Schultheis, P.J., McGill, D.L., Richmond, R.E. & Wagge, J.R. Magnesium deficiency impairs fear conditioning in mice. *Brain Res* **1038**, 100-106 (2005).
35. Serita, T., *et al.* Dietary magnesium deficiency impairs hippocampus-dependent memories without changes in the spine density and morphology of hippocampal neurons in mice. *Brain Res Bull* **144**, 149-157 (2019).
36. Tsuji, R., Inoue, H., Uehara, M. & Kida, S. Dietary magnesium deficiency induces the expression of neuroinflammation-related genes in mouse brain. *Neuropsychopharmacol Rep* **41**, 230-236 (2021).
37. Maier, J.A.M., Locatelli, L., Fedele, G., Cazzaniga, A. & Mazur, A. Magnesium and the Brain: A Focus on Neuroinflammation and Neurodegeneration. *Int J Mol Sci* **24** (2022).
38. Landfield, P.W. & Morgan, G.A. Chronically elevating plasma Mg^{2+} improves hippocampal frequency potentiation and reversal learning in aged and young rats. *Brain Res* **322**, 167-171 (1984).
39. Wang, P., *et al.* Magnesium ion influx reduces neuroinflammation in Abeta precursor protein/Presenilin 1 transgenic mice by suppressing the expression of interleukin-1beta. *Cell Mol Immunol* **14**, 451-464 (2017).
