# Supplementary material for: Intracellular magnesium optimizes transmission efficiency and plasticity of hippocampal synapses by reconfiguring their connectivity
Source: Nat Commun. 2024 Apr 22;15:3406. doi: 10.1038/s41467-024-47571-3 (PMC11035601; doi:10.1038/s41467-024-47571-3)
Supplement: Supplementary file 3 — Reporting Summary [file 41467_2024_47571_MOESM3_ESM.pdf]

nature portfolio

Corresponding author(s): Hang Zhou, Guosong Liu

Last updated by author(s): 2024-2-25

Reporting Summary

Nature Portfolio wishes to improve the reproducibility of the work that we publish. This form provides structure for consistency and transparency in reporting. For further information on Nature Portfolio policies, see our Editorial Policies and the Editorial Policy Checklist.

Statistics

For all statistical analyses, confirm that the following items are present in the figure legend, table legend, main text, or Methods section.

n/a	Confirmed	
☐	☒	The exact sample size (*n*) for each experimental group/condition, given as a discrete number and unit of measurement
☐	☒	A statement on whether measurements were taken from distinct samples or whether the same sample was measured repeatedly
☐	☒	The statistical test(s) used AND whether they are one- or two-sided<br>*Only common tests should be described solely by name; describe more complex techniques in the Methods section.*
☐	☒	A description of all covariates tested
☐	☒	A description of any assumptions or corrections, such as tests of normality and adjustment for multiple comparisons
☐	☒	A full description of the statistical parameters including central tendency (e.g. means) or other basic estimates (e.g. regression coefficient) AND variation (e.g. standard deviation) or associated estimates of uncertainty (e.g. confidence intervals)
☐	☒	For null hypothesis testing, the test statistic (e.g. *F*, *t*, *r*) with confidence intervals, effect sizes, degrees of freedom and *P* value noted<br>*Give P values as exact values whenever suitable.*
☒	☐	For Bayesian analysis, information on the choice of priors and Markov chain Monte Carlo settings
☒	☐	For hierarchical and complex designs, identification of the appropriate level for tests and full reporting of outcomes
☒	☐	Estimates of effect sizes (e.g. Cohen's *d*, Pearson's *r*), indicating how they were calculated

Our web collection on statistics for biologists contains articles on many of the points above.

Software and code

Policy information about availability of computer code

Data collection	Olympus FV300 or FV1000 confocal microscopy (version: Fluoview FV300 or FV1000); Zeiss Supra55 scanning electron microscopy; Spinning disk confocal system (Yokogawa) equipped with a high-speed CCD camera (Andor); Custom-made ultrafast imaging system; Noldus Ethovision animal behavior analysis system (Netherlands).
Data analysis	ImageJ (FIJI, v2.3.0/1.53s); Graphpad prism (v8.4.0); Noldus Ethovision (v2008); Caiman in Python version (v1).

For manuscripts utilizing custom algorithms or software that are central to the research but not yet described in published literature, software must be made available to editors and reviewers. We strongly encourage code deposition in a community repository (e.g. GitHub). See the Nature Portfolio guidelines for submitting code & software for further information.

Data

Policy information about availability of data

All manuscripts must include a data availability statement. This statement should provide the following information, where applicable:

- Accession codes, unique identifiers, or web links for publicly available datasets
- A description of any restrictions on data availability
- For clinical datasets or third party data, please ensure that the statement adheres to our policy

All data are available in the main text or the supplementary materials. Source data are provided with this paper.

Research involving human participants, their data, or biological material

Policy information about studies with human participants or human data. See also policy information about sex, gender (identity/presentation), and sexual orientation and race, ethnicity and racism.

Reporting on sex and gender	*Use the terms sex (biological attribute) and gender (shaped by social and cultural circumstances) carefully in order to avoid confusing both terms. Indicate if findings apply to only one sex or gender; describe whether sex and gender were considered in study design; whether sex and/or gender was determined based on self-reporting or assigned and methods used.*<br>*Provide in the source data disaggregated sex and gender data, where this information has been collected, and if consent has been obtained for sharing of individual-level data; provide overall numbers in this Reporting Summary. Please state if this information has not been collected.*<br>*Report sex- and gender-based analyses where performed, justify reasons for lack of sex- and gender-based analysis.*
Reporting on race, ethnicity, or other socially relevant groupings	*Please specify the socially constructed or socially relevant categorization variable(s) used in your manuscript and explain why they were used. Please note that such variables should not be used as proxies for other socially constructed/relevant variables (for example, race or ethnicity should not be used as a proxy for socioeconomic status).*<br>*Provide clear definitions of the relevant terms used, how they were provided (by the participants/respondents, the researchers, or third parties), and the method(s) used to classify people into the different categories (e.g. self-report, census or administrative data, social media data, etc.)*<br>*Please provide details about how you controlled for confounding variables in your analyses.*
Population characteristics	*Describe the covariate-relevant population characteristics of the human research participants (e.g. age, genotypic information, past and current diagnosis and treatment categories). If you filled out the behavioural & social sciences study design questions and have nothing to add here, write "See above."*
Recruitment	*Describe how participants were recruited. Outline any potential self-selection bias or other biases that may be present and how these are likely to impact results.*
Ethics oversight	*Identify the organization(s) that approved the study protocol.*

Note that full information on the approval of the study protocol must also be provided in the manuscript.

Field-specific reporting

Please select the one below that is the best fit for your research. If you are not sure, read the appropriate sections before making your selection.

☒ Life sciences ☐ Behavioural & social sciences ☐ Ecological, evolutionary & environmental sciences

For a reference copy of the document with all sections, see nature.com/documents/nr-reporting-summary-flat.pdf

Life sciences study design

All studies must disclose on these points even when the disclosure is negative.

Sample size	No statistical methods were used to pre-determine sample sizes, but our sample sizes are similar to those reported in previous publications (Slutstky et al., Neuron, 2004, PMID: 15572114; Slutsky et al., Neuron, 2010, PMID: 20152124)
Data exclusions	No data were excluded.
Replication	For animal experiments in Fig. 7, the experiments were replicated on 3 male rats (control) and 4 male rats (MgT group). respectively. For experiments in Supplementary Fig. 9, the experiments were replicated on 8, 10, 11 males rats for the three groups. For synapse imaging experiments, each experimental condition was replicated at least by 3 different biological repeats (coverslips). Biological repeats were from at least 3 batches of primary cultured hippocampal neurons, and each batch were cultured from digested hippocampal CA3-CA1 tissues collected from 5 neonatal (< 48h) animals of both sexes. All attempts at replication were successful.
Randomization	Cultured neurons on coverslips in each batch or experimental animals were randomly allocated to various groups.
Blinding	For behavior tests, EM-based 3D synapse reconstruction, and histology on ultrathin brain slices, the investigators were blinded to the group allocation during data collection and analysis. For synapse imaging experiments, the investigators were not blinded. This doesn't affect the data analysis because the images were analyzed either automatically or semi-manually by the softwares (see methods for details).

Reporting for specific materials, systems and methods

We require information from authors about some types of materials, experimental systems and methods used in many studies. Here, indicate whether each material, system or method listed is relevant to your study. If you are not sure if a list item applies to your research, read the appropriate section before selecting a response.

Materials & experimental systems

n/a	Involved in the study
☐	☒ Antibodies
☒	☐ Eukaryotic cell lines
☒	☐ Palaeontology and archaeology
☐	☒ Animals and other organisms
☒	☐ Clinical data
☒	☐ Dual use research of concern
☒	☐ Plants

Methods

n/a	Involved in the study
☒	☐ ChIP-seq
☒	☐ Flow cytometry
☒	☐ MRI-based neuroimaging

Antibodies

Antibodies used

Rabbit polyclonal anti-ERC1b/2 (ELKS), Synaptic Systems, Cat#143003
Mouse monoclonal anti-GluA2 (clone 6C4), Invitrogen, Cat#32-0300
Guinea pig polyclonal anti-MAP2, Synaptic Systems, Cat#188 004
Mouse monoclonal anti-Munc13-1 (clone 266B1), Synaptic Systems, Cat#126 111
Rabbit polyclonal anti-Munc13-1, Synaptic Systems, Cat#126103
Mouse monoclonal anti-PSD95 (clone 7E3-1B8), Millipore, Cat#CP35
Mouse monoclonal anti-Rab3a (clone 42.2), Synaptic Systems, Cat#107111
Rabbit polyclonal anti-Rab3a, Synaptic Systems, Cat#107102
Rabbit polyclonal anti-RIM1, Synaptic Systems, Cat#140003
Mouse monoclonal anti-Synaptophysin (clone SY38), Millipore, Cat#MAB5258
Guinea pig polyclonal anti-Synaptophysin, Synaptic Systems, Cat#101004
Mouse monoclonal anti-Synaptotagmin1 (clone 41.1), Synaptic Systems, Cat#105011
Mouse monoclonal anti-Syntaxin1 (clone 78.2), Synaptic Systems, Cat#110011
Guinea pig polyclonal anti-VGLUT1, Millipore, Cat#AB5905

Validation

These antibodies have been well validated by manufacturers and in published articles. Websites: https://www.sysy.com, https://www.thermofisher.cn/cn/zh/home/brands/invitrogen.html.html, https://www.emdmillipore.com. References, PMID: 26184109, 15572114, 20152124.

Animals and other research organisms

Policy information about studies involving animals; ARRIVE guidelines recommended for reporting animal research, and Sex and Gender in Research

Laboratory animals

Sprague-Dawley rats were purchased from Vital River Laboratory (Beijing, China) or Anhui Medical School Animal Facility (Hefei, China).

Wild animals

No wild animals were used in the study.

Reporting on sex

For experiments in Fig. 7 and Supplementary Fig. 9, the animals used were all male. For other exepriments, neonatal rats of both sexes were used.

Field-collected samples

No field-collected samples were used in the study.

Ethics oversight

All animal experiments were carried out in accordance with institutional guidelines and were approved by the Institutional Animal Care and Use Committee (IACUC) at Tsinghua University, China, and the IACUC at University of Science and Technology of China.

Note that full information on the approval of the study protocol must also be provided in the manuscript.

Plants

Seed stocks

Report on the source of all seed stocks or other plant material used. If applicable, state the seed stock centre and catalogue number. If plant specimens were collected from the field, describe the collection location, date and sampling procedures.

Novel plant genotypes

Describe the methods by which all novel plant genotypes were produced. This includes those generated by transgenic approaches, gene editing, chemical/radiation-based mutagenesis and hybridization. For transgenic lines, describe the transformation method, the number of independent lines analyzed and the generation upon which experiments were performed. For gene-edited lines, describe the editor used, the endogenous sequence targeted for editing, the targeting guide RNA sequence (if applicable) and how the editor was applied.

Authentication

Describe any authentication procedures for each seed stock used or novel genotype generated. Describe any experiments used to assess the effect of a mutation and, where applicable, how potential secondary effects (e.g. second site T-DNA insertions, mosiacism, off-target gene editing) were examined.
